# To Our Readers

**Published:** 1883-10

**Authors:** 


### HALL'S JOURNAL OF HEALTH

AND

### MISCELLANY.

MONTHLY.

E. H. GIBBS, A. M., M. D., EDITOR.

FOUNDED IN 1854.

### TO OUR READERS.

THERE is no treasure so precious as health, and none so wickedly squandered. The negligences of the majority; their ignorance in accidents, emergencies and sickness, reduces the average term of life one-half. Millions of useful lives are lost for want of a few simple rules for the care of the health that this journal gives in language easily understood.

The most absurd objection to "health rules" is that their observance is more of a burden than disease itself. The answer is that the "rules of health" are few and simple; and that their observance soon becomes easier than their violation; that good health not only qualifies us for the active duties of life, but causes us to enjoy its pleasures with a keener relish.

In a religious point of view health is a duty; and sickness induced by self-neglect is a sin.

The great mortality among little children is one of the saddest features of modern civilization.

This destruction of the "innocents" occurs to a greater extent among the poor and degraded; then the wonder is that any escape; but in the higher walks of life death also comes to children to an alarming extent. There is no valid reason why children who are born with good constitutions should die in infancy. The cause is not difficult to find. It is owing to the ignorance of most mothers of what is essential to the health of their offspring as relates to food, clothing and proper care. How many mothers are there in any class of society to-day, or, to make the question broader, how many persons outside of the medical profession would be able to prevent bleeding to death if an important artery had been severed, or to save the life of a person who had swallowed a fatal dose of poison, or a child suffocating from croup? Not one out of ten, it is safe to say, though five of the ten would probably be ashamed to confess their ignorance of the last popular novel, or of what is being done in the theatrical world. The most fatal disease among children is *cholera infantum*," and yet no child ever died of that disease who was promptly and properly treated. The same is true to a great extent of other diseases.

The JOURNAL has labored for thirty years to induce people to live properly, that their lives might be lengthened to the reasonable term of human existence. Its efforts have been appreciated by the thoughtful few, but regarded with utter indifference by the vast majority. We have always felt that to urge people to subscribe for HALL'S JOURNAL OF HEALTH is a good deal like urging them to change their natures. A majority are improvident and reckless by habit which becomes second nature; and thus they perish before their time. We do not expect large accessions to our subscription list from this class; it is unfortunately true that patrons of health journals are usually persons who *could* afford to dispense with that kind of reading—if indeed any one could—while those who need such instruction most don't appreciate it.

We promise to make the JOURNAL more attractive than ever, by publishing articles that will be of interest to all and especially the family. The 12 monthly numbers for 1884 will make a volume of unusual interest as a book of reference, as it will describe all the ordinary diseases to which persons, and particularly children, are liable. The first symptoms of these diseases will be described in plain terms and advice as to the best methods of treatment. Particular attention will be given to the methods to be employed in accidents and emergencies. The subscription price of HALL'S JOURNAL OF HEALTH is $1 a year. News Agents and Booksellers will be allowed a commission of 25 cents for each subscription sent us. Any postmaster who sends us the name of one yearly subscriber, with $1, will himself receive the JOURNAL one year FREE. For each additional subscriber he will be entitled to the usual commission of 25 cents.

In looking for a premium for subscribers we have unhesitatingly selected the NATIONAL STANDARD DICTIONARY as a most desirable book. A reliable dictionary is indispensable to every one. The wonder is how a dictionary containing 40,000 words—and in addition to this 700 illustrations, a Biographical Register, foreign phrases, geographical names, weights and measures of various nations, Mythological Dictionary and much other valuable information—can be handsomely bound and sold for $1, at which price we will send the Dictionary, post-paid, to any address in the United States or Canada. Or we will send the Dictionary and also HALL'S JOURNAL OF HEALTH one year for $1.50.

That popular book of family medicine, DR. HALL'S HEALTH AT

HOME, bound in cloth, we will send by mail, post-paid, on receipt of the regular price—$4.00—and we will in addition send HALL'S JOURNAL OF HEALTH, *free*, for one year to the purchaser, or to any other address desired. Or, we will send the NATIONAL STANDARD DICTIONARY, DR. HALL'S HEALTH AT HOME, bound in cloth, and HALL'S JOURNAL OF HEALTH one year—all for $4.50. If leather binding is required for HEALTH AT HOME, 40 cents extra must be sent us. Remit by postal notes.

P. S.—Those who subscribe for the JOURNAL OF HEALTH for 1884 before Dec. 2 will receive the numbers for November and December, 1883, FREE. Address E. H. GIBBS & Co.,

Publishers of HALL'S JOURNAL OF HEALTH,
New York City.

